# Supplementary material for: Profiling of metabolites, proteins, and protein phosphorylation in silica-exposed BEAS-2B epithelial cells
Source: PLoS One. 2023 Apr 11;18(4):e0273034. doi: 10.1371/journal.pone.0273034 (PMC10089310; doi:10.1371/journal.pone.0273034)

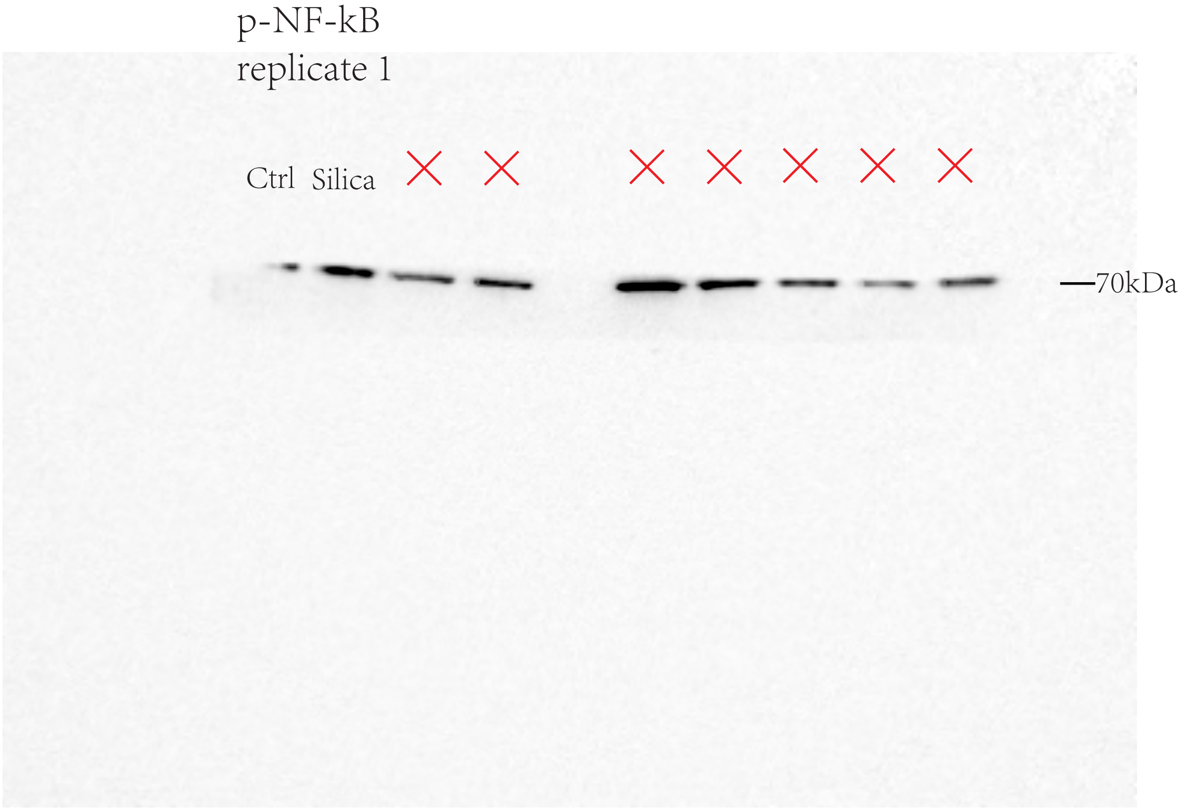
p-NF-kB
replicate 1
Ctrl
Silica
70kDa

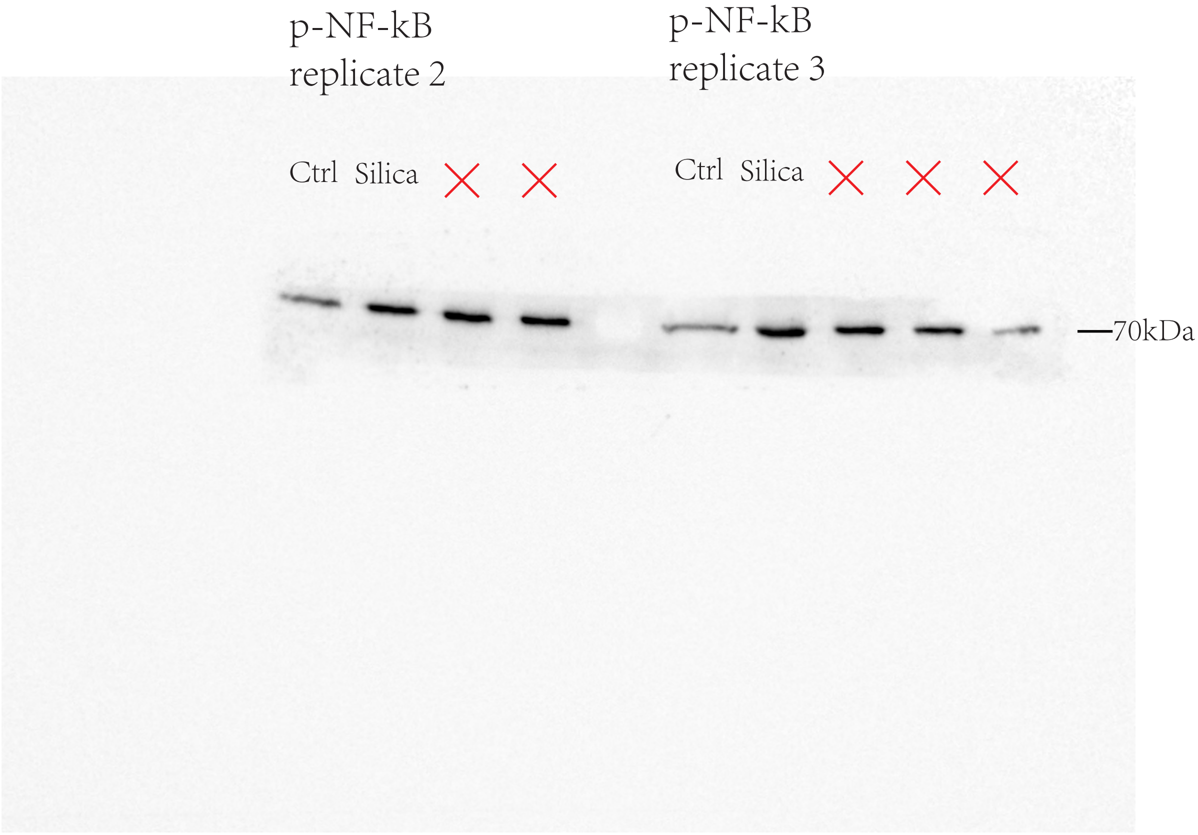
p-NF-kB
replicate 2
p-NF-kB
replicate 3
Ctrl
Silica
Ctrl
Silica
70kDa

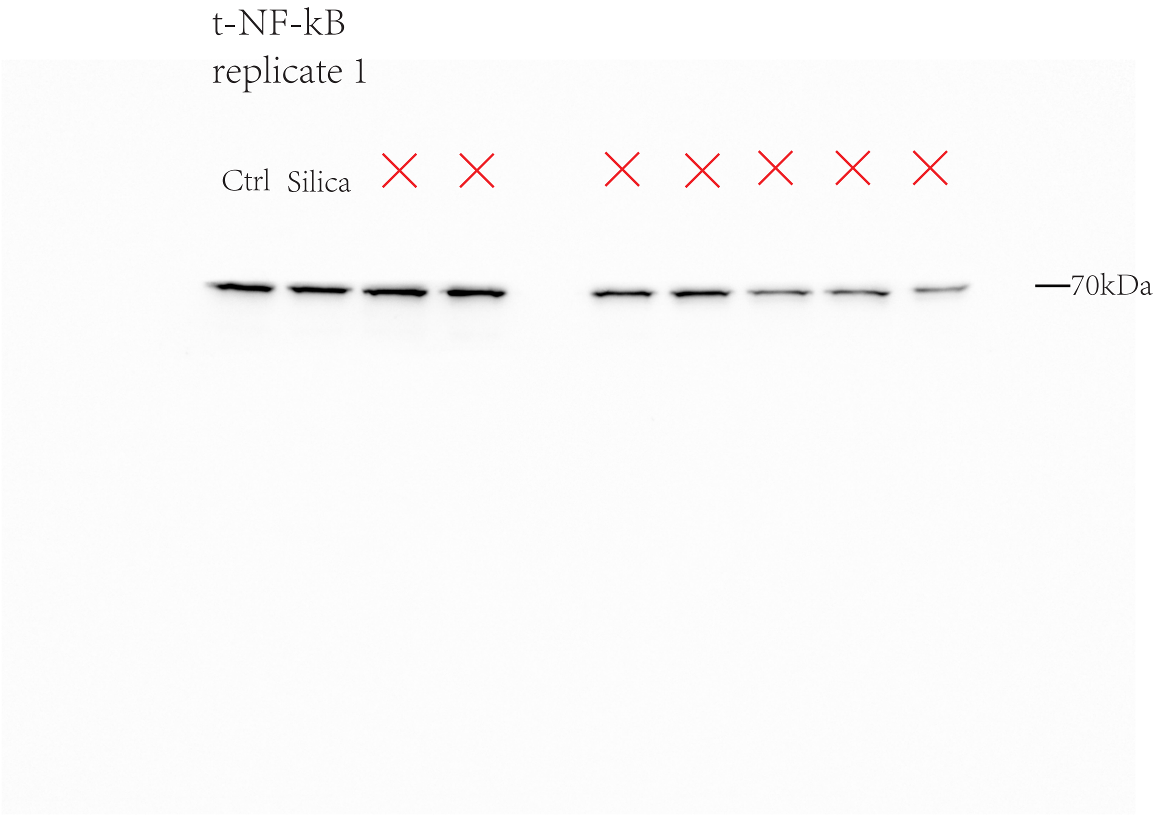
t-NF-kB
replicate 1
Ctrl
Silica
70kDa

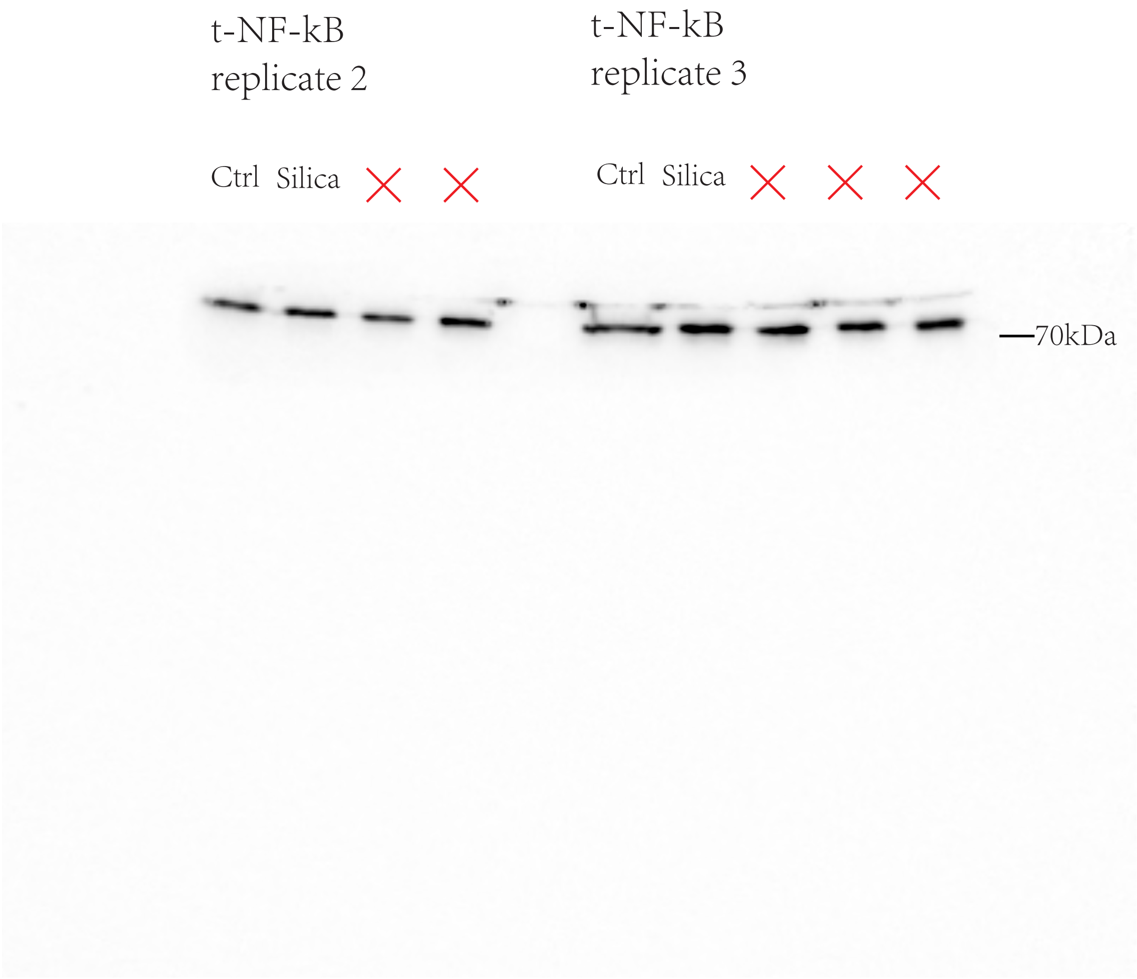
t-NF-kB
replicate 2
t-NF-kB
replicate 3
Ctrl
Silica
Ctrl
Silica
70kDa

actin
replicate 1

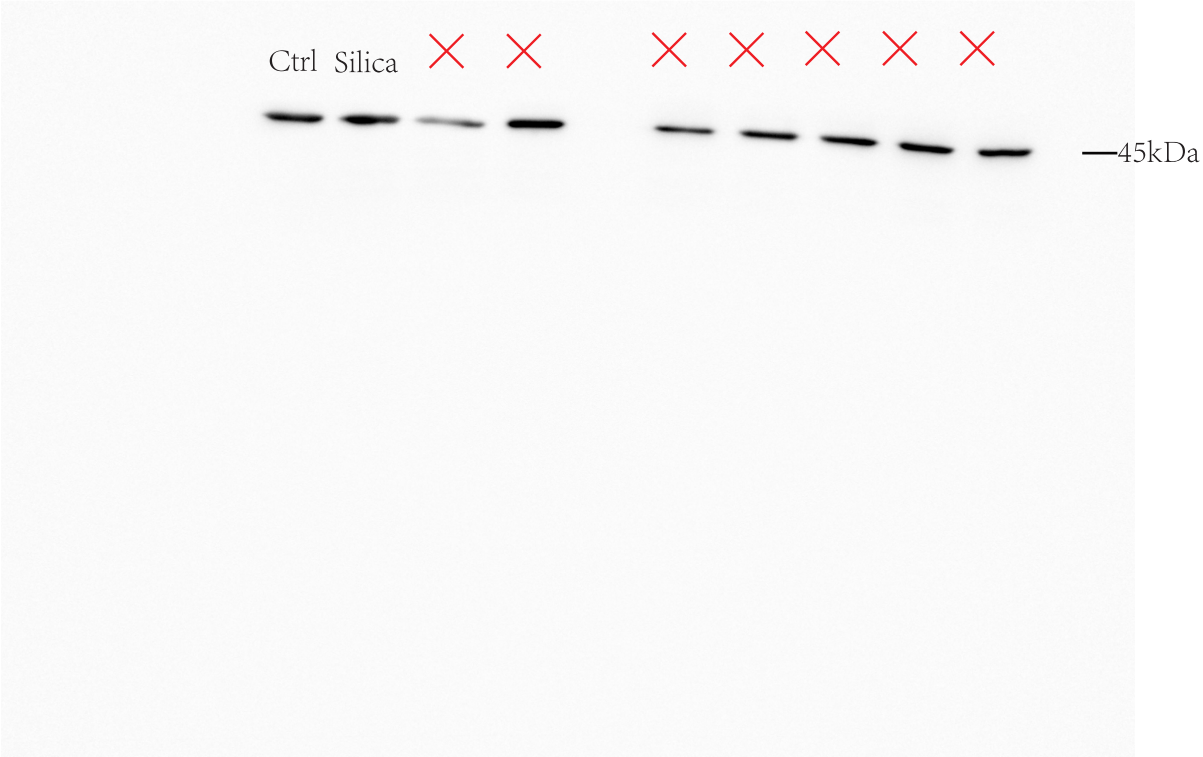

actin
replicate 2

actin
replicate 3

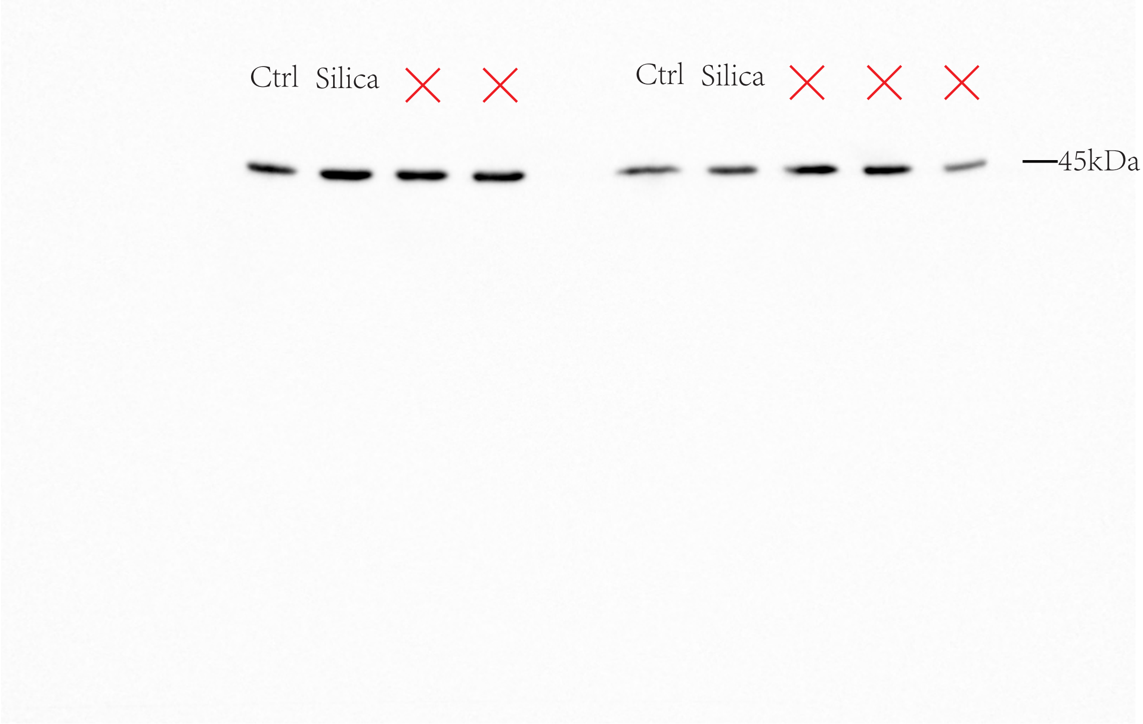

Supplement: S1 Raw image — (PDF) [file pone.0273034.s005.pdf]
